# Learning and navigating digitally rendered haptic spatial layouts

**DOI:** 10.1038/s41539-023-00208-4

**Published:** 2023-12-16

**Authors:** Ruxandra I. Tivadar, Benedetta Franceschiello, Astrid Minier, Micah M. Murray

**Affiliations:** 1 The Department of Radiology, Lausanne University Hospital and University of Lausanne, Lausanne, Switzerland; 2 Department of Ophthalmology, Fondation Asile des Aveugles, Lausanne, Switzerland; 3 Centre for Integrative and Complementary Medicine, Department of Anesthesiology, Lausanne University Hospital and University of Lausanne, Lausanne, Switzerland; 4 Cognitive Computational Neuroscience Group, Institute for Computer Science, University of Bern, Bern, Switzerland; 5 The Sense Innovation and Research Center, Lausanne and Sion, Switzerland; 6 Institute of Systems Engineering, School of Engineering, University of Applied Sciences Western Switzerland (HES-SO Valais), Sion, Switzerland

**Keywords:** Perception, Human behaviour

## Abstract

Learning spatial layouts and navigating through them rely not simply on sight but rather on multisensory processes, including touch. Digital haptics based on ultrasounds are effective for creating and manipulating mental images of individual objects in sighted and visually impaired participants. Here, we tested if this extends to scenes and navigation within them. Using only tactile stimuli conveyed via ultrasonic feedback on a digital touchscreen (i.e., a digital interactive map), 25 sighted, blindfolded participants first learned the basic layout of an apartment based on digital haptics only and then one of two trajectories through it. While still blindfolded, participants successfully reconstructed the haptically learned 2D spaces and navigated these spaces. Digital haptics were thus an effective means to learn and translate, on the one hand, 2D images into 3D reconstructions of layouts and, on the other hand, navigate actions within real spaces. Digital haptics based on ultrasounds represent an alternative learning tool for complex scenes as well as for successful navigation in previously unfamiliar layouts, which can likely be further applied in the rehabilitation of spatial functions and mitigation of visual impairments.

## Introduction

It has been recently shown that digitally simulated haptics can confer spatial object relations by reducing screen friction via ultrasonic feedback in both sighted and visually impaired individuals^1–3^. The technology that we refer to here as “digital haptics” operates in the following manner. The touchscreen of a tablet device monitors the position of the fingertip. Localised piezoelectric actuators, in turn, vibrate at ultrasonic frequencies to dynamically change the perceived friction at the fingertip and thus give the impression of texture. Specifically, our laboratory has tested the efficacy of this technology during haptically-based object recognition and mental rotation. In Tivadar et al. (2019, 2020) blindfolded sighted and visually impaired participants were asked to feel ultrasonic vibrotactile letters displayed digitally on the screen of a haptic tablet (i.e., surface haptic display^4^, or digital interactive map^5^) presented at various orientations (i.e., rotated at 0, 90, 180, or 270°) via active exploration with the fingertip. They then decided whether these letters were presented in mirror-reverse or normal form. To complete the task, participants had to first recognise the letter, then resolve the orientation, rotate it in their minds back to a 0° angle, and then realise whether the image is presented in normal or mirror-reverse. Participants were able to successfully use the digital haptics in this mental rotation task of 2D letter stimuli, demonstrating that digital haptics can support both the creation of mental images, and the mental manipulation of these images. In addition, a multitude of recent studies has investigated the use of vibrotactile cues to transmit graphical and spatial information and to support spatial tasks such as object recognition, detection of orientation, and creation of cognitive maps and wayfinding (reviewed in^6^, see also^7^). However, it remains unknown whether spatial information can also be transmitted by technology manipulating skin indentation using ultrasonic tactile feedback.

Spatial functions related to mobility and navigation can be supported by visual, tactile, and auditory stimuli^8–11^; see ref. ^11^ for a recent review). Despite the fact that vision and touch use different metrics and geometries^12^, and that there is no one-to-one mapping between them, vision and touch are intimately linked^13^. For example, visual areas are activated during a variety of tactile tasks^14^, for example, during perception of haptic/tactile form^15–18^. In addition, vibrotactile cues have proven efficient in capturing spatial attention^19^ and guiding visual search performance^20^. While visual experience might be necessary to develop the brain areas supporting normal spatial functions^21^, research demonstrates that spatial representations can be achieved in a largely modality-independent fashion^22^, and engage a common representational system^8,23^. Indeed, the functional equivalence hypothesis states that processes that depend only on spatial images as their positional inputs will treat in an equivalent manner a spatial image from different senses, as well as a spatial image from language, which occupy the same location in representational space and has the same degree of precision^24,25^. According to this hypothesis, while visual and auditory processing result directly in spatial percepts of the visual and auditory stimuli, linguistic stimuli confer meaning which can, upon further processing, also give rise to the putative spatial image.

Spatial learning in humans is primarily supported by the hippocampal-entorhinal formation^26^. Using spatial memory, we encode our environment and spatial relationships within it into cognitive maps, which guide spatial navigation^27^. Spatial navigation makes use of allocentric (i.e., world-centred) and egocentric (i.e., body-centred) spatial representations, or cognitive maps. The hippocampal-entorhinal formation together with parietal cortical structures store cognitive maps for different environments and reference frames as well as altered versions of the same environment^28^ using detailed individual representations^29^ as well as generalisable codes^30^. Spatial learning is thought to rely on a mechanism of replay and pre-play of firing sequences^31^, which enables memory consolidation. Spatial navigation as a process is thought to rely on concerted activity between many regions. Specifically, it is hypothesised that the posterior parietal cortex integrates perceived spatial orientation, involving the perception of the organisms’ current location and directional heading within its environment, with the general spatial view of the world, involving spatial relationships of landmarks with the aim of route or trajectory formation^32^. This intricate mapping mechanism is thought to support spatial learning and navigation, which is ultimately based on cognitive spatial maps.

Spatial relations can be conveyed using digital technology in sensory substitution devices^1,3,6,33–37^. For example, tactile maps have long been recognised as a useful tool in mobility training^38^, their main consumers being the visually impaired and blind populations^39^. However, maps are static, cumbersome to create and have many associated cartographic problems^40^. Touch poses some disadvantages when compared to sight, namely a lower resolution of the fingertip compared to the eye and a limited serial perception^40^. Recently, the research on tactile maps concluded that on the whole, tactile mapping remains a specialist subject, as well as being expensive, hard to obtain, difficult to make and unable to match a visual map’s resolution, even using digital interactive technologies^41^. In addition, cartographers, who are usually sighted, are faced with the problems of simplification, generalisation, classification, and symbolisation to render a visual map tactile^40^. Moreover, tactile map design is largely based upon individual requirements, leading to the production of bespoke maps dependent upon the knowledge, experience, and skills of intended single users^42^. Further research and development in the field are required to overcome current limitations and obstacles to practicality. In particular, novel technologies that allow for digital rendering of tactile information (e.g., digital haptic tablets such as the Xplore Touch, www.hap2u.net), promise to open up the world for patients due to the ease of digital information processing and transfer. Digital vibrotactile maps provide use-case flexibility for users^43^, can be dynamic, multimodal, and produced on portable platforms^6^. Specifically, vibration, audio and kinaesthetic feedback can render dense and complex map information^43^. This multimodality, in turn, can lead to improved learning^44^. While tactile maps were previously restrained to expensive pin arrays and force-feedback devices, the technology is now available on smartphones and tablets. Indeed, replacing tactile stimulation, such as Braille, with audio-tactile interactions, improved efficiency and user satisfaction^44^. Such innovative developments in technologies employed for tactile sensory substitution devices nevertheless suffer from some disadvantages that prevent them from reaching the mass market. Specifically, such devices lack ergonomics, accessibility, and require high investment of resources, such as time, money, and medical personnel^45–47^.

Previous research has shown that vibro-audio maps can be as efficient as an analogous hardcopy tactile map^6^ in supporting the creation and manipulation of spatial cognitive maps, and in assisting wayfinding. Such functions have also been shown to be supported by other touchscreen-based vibrotactile stimulation. However, it is yet unknown whether haptics alone can support these functions via feedback using ultrasonic vibration. Also, the majority of studies testing spatial functions such as navigation and cognitive map creation using vibrotactile stimuli focus on blind and visually impaired participants (reviewed in ref. ^6^) and less on sighted participants. To investigate how sighted participants create and manipulate cognitive maps and then use these in navigation, we tested normally-sighted blindfolded participants on a trajectory navigation task, during which the layout’s outline topologies were presented solely in vibrotactile haptic form. Participants were required to mentally encode and then physically navigate trained and untrained trajectories through a real-world spatial layout (i.e., a living lab apartment). We recorded participants’ behaviour and their understanding of the space (through reconstructions using LEGO^®^ toys) to better assess how users’ interaction with this novel technology develops as a function of training. We also measured how well participants learned to navigate using the haptic tablet, by measuring how well they performed on trajectories that were previously trained, compared to previously untrained routes. We expected participants to be able to learn the spatial layouts to reconstruct them, and also to navigate trajectories in the real space they represented. Specifically, we hypothesised that participants’ spatial outline reconstruction would reach a high degree of similarity with an ideal reconstruction after training, and that their performance on and exploration of trained trajectories would exceed those on untrained trajectories.

## Results

**Fig. 1:**
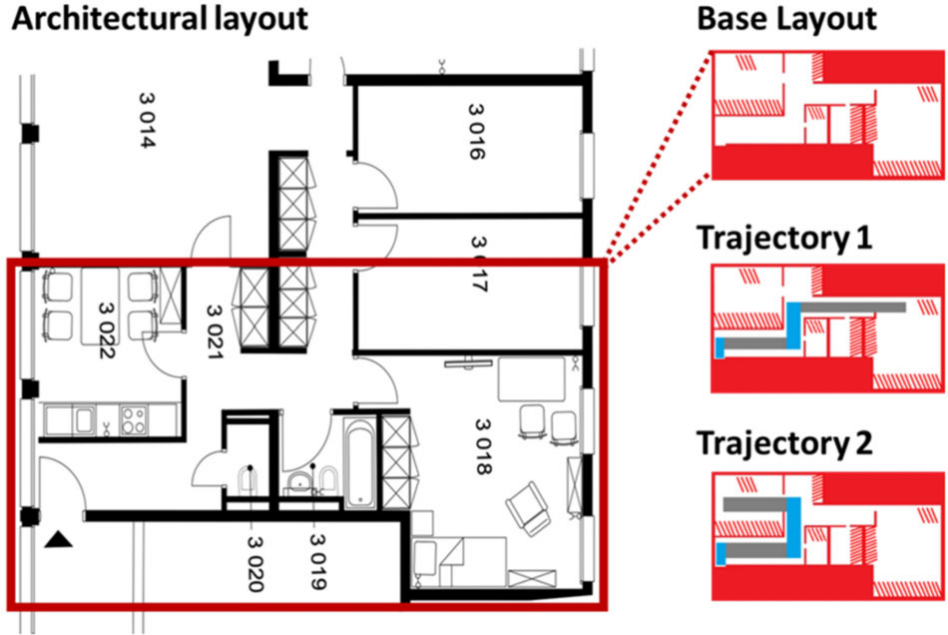
Layouts for the living lab apartment. Based on architectural plans (left), layouts were created (right). The differently disposed red objects (i.e., diagonal lines) represent furniture obstacles, while the fully coloured rectangles represent areas to be ignored, and the red simple lines represent walls. White areas were void of any texture. The two trajectories to be explored by subjects are displayed. The light blue and grey colours represented the same texture, oriented differently (i.e., horizontally vs. vertically) in order to confer the same sensation.

**Fig. 2:**
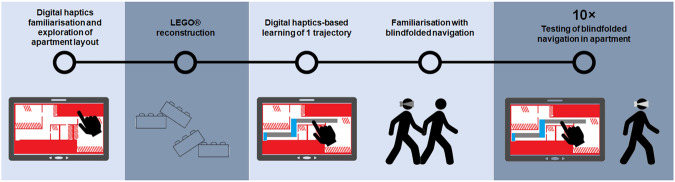
Schematic of the experimental procedure. All training steps are illustrated on a light blue background, whereas the testing steps are indicated by a dark blue background. Blindfolded participants first explored the layout of the apartment, which was rendered in haptic form on the tablet. Next, blindfolded participants reconstructed this learned layout using LEGO® pieces and board. Blindfolded participants were then trained on a certain trajectory and on blindfolded navigation, after which they were tested on independent blindfolded navigation in the apartment following either the trained or untrained trajectory.

As described in the Methods section at the end of the manuscript, participants were blindfolded and wore noise-canceling headphones throughout the whole duration of the experiment. They were required to feel 2D layouts of a real space (Fig. 1) presented on the digital haptics tablet. To this end, participants were familiarised with haptic textures and taught how to explore the haptic tablet. After the encoding phase with the digital haptics tablet, participants had to reconstruct the full spatial layout using LEGO^®^ bricks. As a next step, participants were familiarised with the trajectory texture, and training on a certain trajectory in the previously learned 2D space continued. Participants were finally familiarised with the real space (i.e., the apartment) they had to sightlessly navigate. To this end, they were taken inside the apartment by the experimenter, where they navigated the trained trajectory and learned about the error zones, and what would be counted as an error during the testing phase. Finally, participants were tested on their navigation. Here, participants were asked to independently explore the tablet and then physically complete the presented trajectory to end in either Room1 or Room2 (We would note that this aspect of the destination room was not communicated to the participants). This phase was comprised of 10 trials (i.e., 10 completed trajectories), split between 5 repetitions of each of 2 trajectories (one trained and one untrained) that were randomised for each participant. The overall procedure is schematized in Fig. 2. GoPro cameras were mounted onto participants’ heads and filmed their maze exploration.

## Inter-rater reliability

### LEGO® reconstructions

The photos of the LEGO® reconstructions were first submitted to a subjective rating procedure involving 3 independent raters. Inter-rater reliability (IRR^48^) was assessed for consistency and agreement using 2 two-way mixed, consistency/agreement, average-measures intraclass correlation (ICC)^49^. The resulting ICCs (*ICC*_*c*_ = 0.863, *ICC*_*a*_ = 0.826) were in the excellent range^50^, indicating that the three raters had a high degree of agreement and consistency amongst them. These ICCs values suggest that a minimal amount of measurement error was introduced by the independent raters.

### Behaviour

As the behavioural data during the navigation phase of the protocol (see section *Behaviour* below) were also rated by two independent individuals, IRR was assessed. IRR for accuracy scores (*κ* = 1, *p* < 0.01), indicated perfect agreement^51^. Agreement for IRR was assessed using a two-way mixed, consistency, average-measures ICC^49^ to assess the degree that coders provided consistency in their ratings of Errors and Time off Track across participants. The resulting ICC was in the good range for Errors, ICC_Err_ = 0.64, and in the excellent range for Time off Track, ICC_ToT_ = 0.76^50^, indicating that coders had a fairly high degree of agreement and suggesting that coders rated the values similarly. The fairly high ICCs and kappa values suggest that a minimal amount of measurement error was introduced by the independent coders, and therefore statistical power for subsequent analyses is not substantially reduced.

## LEGO® reconstructions

**Fig. 3:**
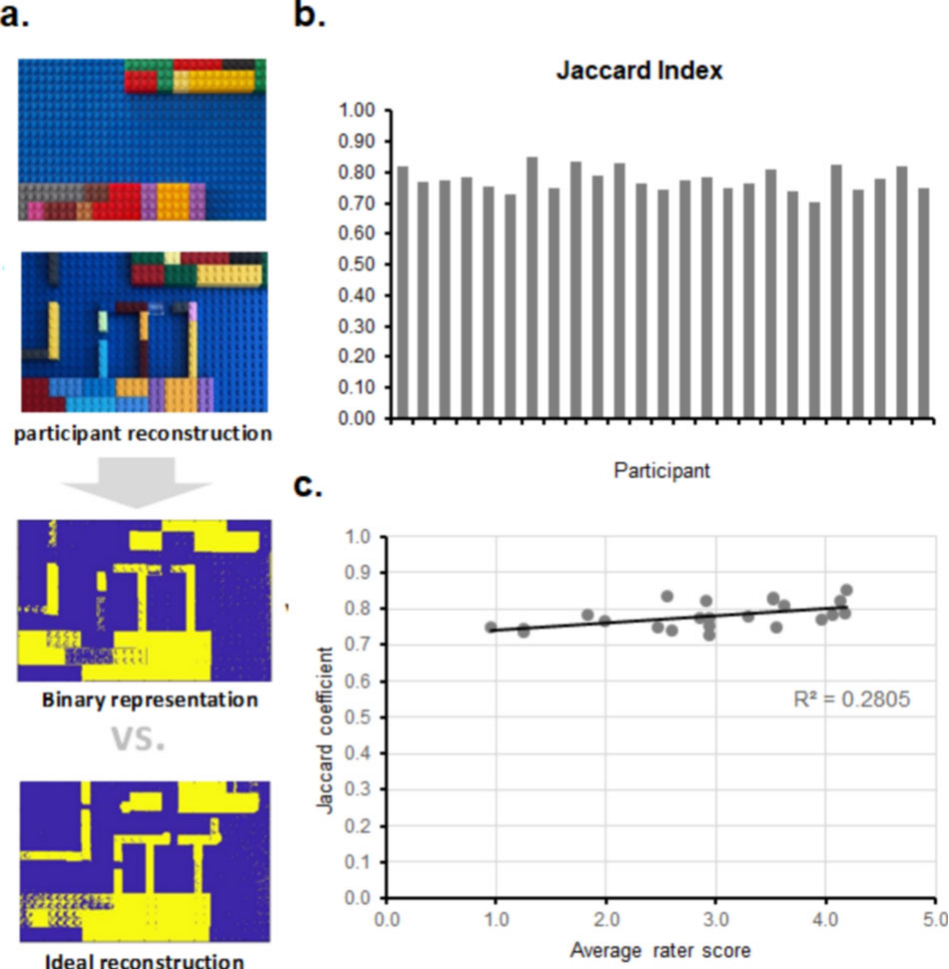
LEGO^®^ reconstruction analysis procedure and results. **a** Exemplar Procedure for computing the Jaccard index. The plain background LEGO® board. The Jaccard index was computed by taking photographs of participants’ LEGO® reconstructions, turning them into binary representations, and comparing them to an ideal reconstruction. **b** The Jaccard index from each participant. **c** Scatterplot of the Jaccard index vs. mean rater scores as well as the linear regression of the data (i.e., Pearson’s correlation with the *R*^2^ value indicated in the inset).

We thus took the mean of the ratings across the three raters and compared this to the Similarity Index, which we computed by comparing binarized photographic renderings of the LEGO® reconstruction to a binarized photographic rendering of an ideal reconstruction (Fig. 3). We observed a significant correlation (measured as a Pearson’s *r*) between the Similarity index and these mean scores of the subjective ratings (*r* = 0.53, *t*_(23)_ = 2.99, *p* = 0.006; 95% confidence Interval [0.17; 0.77]), after ensuring that these were normally distributed using the Shapiro-Wilk test. These high Similarity Indices (all above 0.7) that we validated through correlations with the subjective ratings provide an indication that participants were able to learn the layout of the space from the haptic tablet and to physically reconstruct it in the absence of any external visual input.

## Behaviour on trajectories

**Fig. 4:**
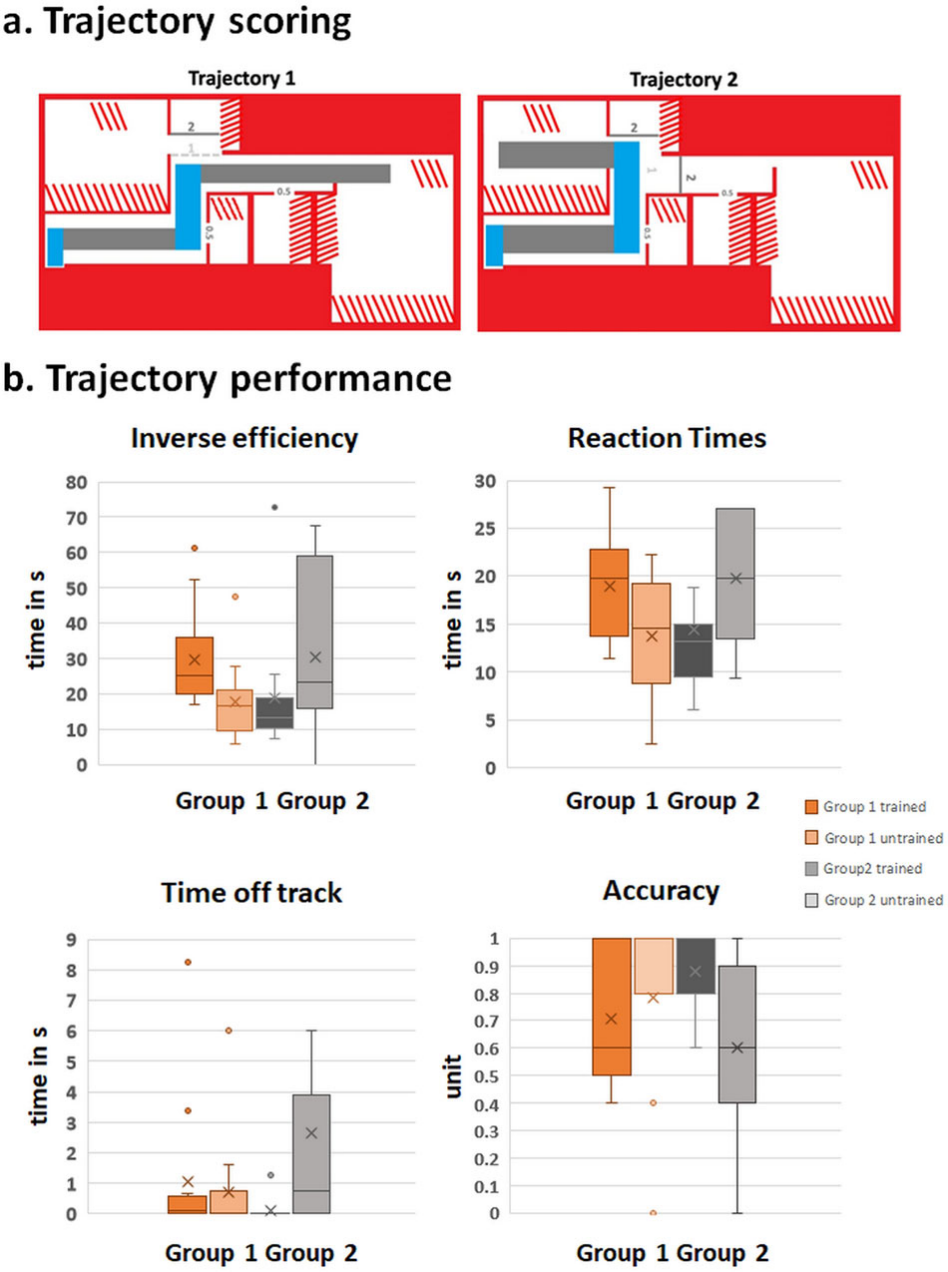
Scoring and performance measures of trajectory navigation based on learning from digital haptics. **a** Trajectory scoring. The numbers represent areas “off track” for each of the trajectories and index the number of error points participants received when exploring those areas. For example, if a participant touched a door to another room and thus used it as a landmark, they would get 0.5 error points. **b** Trajectory performance. Inverse Efficiency scores and Reaction Times scores are displayed on the upper half, while Time off Track and Accuracy are shown on the lower half, with Group 1 and Group 2 representing the groups (i.e., Group 1 trained on the harder trajectory, in orange; Group 2 trained on the easier trajectory, in grey). Darker colours illustrate trained and lighter colours untrained mazes. The box and whisker plots show the mean (denoted by “*x*”), the median, (denoted by the horizontal line), the interquartile range (denoted by the vertical extent of the box), any variability outside this range (denoted by the whiskers), as well as any outliers (denoted by the points).

In terms of behavioural performance, we assessed five dependent variables: Accuracy (Acc), Reaction Times (RT), Tablet Exploration Time (ET), Errors (E), and Time off Track (Toff). Outliers were calculated on a single-subject basis (i.e., by subject and trajectory). However, outlier exclusion processes resulted in no outliers being excluded. Means for each dependent variable were calculated, and repeated-measures permutation ANOVAs, using 5000 permutations, were run with the within-subjects factor Training (whether the trained vs. untrained trajectory was tested) and the between-subjects factor Group (Group 1 trained on the more difficult trajectory, Group 2 trained on the easier trajectory). Paired two-sided permutation t-tests with 5000 permutations were run as post-hoc comparisons. Results are illustrated in Fig. 4. The seed was set at 42. A significant interaction was observed for Accuracy (*p* = 0.004, *pω*^2^ = 0.14). Data were further split by Group, and post-hoc comparisons were run between trained and untrained trajectories. While there was no significant difference for Group 1, the permutation t-test was significant for Group 2 (*t* = −3.44, *p* = 0.01, *d* = −0.95), with higher accuracy for trained trajectories than untrained trajectories (0.88 vs. 0.57, respectively). The Training × Group interaction was also significant for Toff (*p* = 0.02, *pω*^*2*^ = 0.06). Permutation t-tests with the factor Training showed a significant difference for Group 2 (*t* = 2.12, *p* = 0.003, *d* = −0.59), with more time spent off track on untrained than trained trajectories (2.7 s vs 0.1 s), while no significant difference between trained and untrained trajectories was found for Group 1. The Training × Group interaction was also significant for RTs (*p* = 0.01, *pω*^*2*^ = 0.11). The post-hoc permutation *t* tests were significant only for Group 1 (Group 1: *t* = −2.95, *p* = 0.01; *d* = −0.81, mean trained: 18.93; mean untrained: 13.71 seconds). A significant Training × Group interaction was also observed for IE scores (*p* = 0.008, *pω*^*2*^ = 0.17). Post-hoc Wilcoxon tests compared trained vs untrained trajectories for Group 1 and Group 2. The tests were significant for Group 1: *t* = −5.7015, *p* = 0.0008, *d* = −1.58; mean trained trajectories: 29.6, mean untrained trajectories: 17.7), but not Group 2.

Furthermore, to test whether there were any associations between the trajectory learning phase based solely on digital haptics and behavioural performance during trajectory completion, we ran correlation analyses between the Jaccard indices and significant behavioural variables for trained and untrained trajectories and each group independently using Kendall’s tau correlation coefficient and False Rate Discovery (FDR) correction for multiple tests. While there were no significant correlations for Group 1 for either the trained or untrained trajectories with the Jaccard index, we found a significant negative correlation between Jaccard and IE scores for Group 2 on untrained trajectories (*τ* = −0.63, *p* = 0.04). However, FDR correction nevertheless resulted in inflation of the *p* value (*p* = 0.14).

## Discussion

Our results provide the first demonstration that vibrotactile cues conveyed via friction reduction using ultrasonic vibration on digital displays allow individuals to learn layouts and spatial navigation paths within complex environments, such as an apartment. Participants’ performance proves the efficacy of ultrasonic digital haptic technologies for transmitting information that supports topographical mapping of both mental representations and their reconstructions as well as navigation within real-world spatial layouts. After only 45 minutes of training on a haptic rendering of a 2D-layout of a real space, participants were able to acquire mental images of that space and reconstruct these images using LEGO® pieces, reaching high similarity when compared to an ideal reconstruction. In addition, these sighted blindfolded participants were able to individually read trajectories and navigate learned spaces after only a short familiarisation trial run where they received sightless navigation help from trained experimenters. Participants performed generally well on easy trajectories. However, participants who were trained first on easy trajectories performed worse on untrained harder trajectories compared to participants who had been trained on these harder trajectories, who then performed very well on the easier trajectory. The only difference between these two groups was the time that people spent on the trajectories, with participants trained on harder trajectories taking more time to navigate these than the easy trajectories, whereas no difference was observed for participants trained on the easy trajectories in their navigation of both hard and easy mazes, except that they spent more time off track on the hard, untrained trajectories.

Thus, using digital haptics, we tested whether blindfolded sighted individuals would be able to learn 2D layouts of completely unknown real-life spaces and then be able to navigate these spaces based only on their learning of haptic rendering. The first measure indexing the amount of learning was participants’ ability to reconstruct the layout of the apartment, whereas the second was how well participants actually performed during navigation of the trajectories. This ability to reconstruct the trajectories was measured as a similarity index between these reconstructions and an ideal reconstruction. The Jaccard index is a common measure in image processing that has previously been used to rate the amount of shared features in problems such as assessing the similarity of sets of patterns^52^ or RNA cells within a cluster^53^. The Jaccard index ranges from 0 to 1, 0 indicating no agreement while 1 indicates perfect similarity. All of the Jaccard indices in our participant group surpassed 0.7, indicating high agreement with our ideal reconstruction. We validated these scores by having 3 independent raters, two of whom were double-blinded, rate the correctness of the reconstructions. These two scores (i.e., means of raters’ scores vs the similarity index) were moderately but significantly correlated, providing validity to the Jaccard ratings. We cannot completely exclude that the verbal indications that participants received throughout the layout learning session to teach them how to interact with the haptic technology had an impact on the mental spatial images that participants created, despite our best efforts not to give any quantitative feedback. When verbally describing the space at the end of the training session, participants were only confirmed or informed regarding their appreciation of the number of rooms available in the apartment and the general shape of the corridor and received encouragements to explore further. While language and semantic stimuli are able to confer spatial relations, these are more qualitative and categorical, and language lacks the metric precision usually available through vision^24,25^. However, it is largely considered that perceptual and language spatial representations are functionally similar^24^. Indeed, people who learn spatial layouts from verbal descriptions or maps build equivalent spatial mental images^54^. However, the functional equivalence hypothesis^24,25^ posits that in order to support spatial imagery, verbal information needs to be as precise as information from other senses (i.e., verbal instructions should be as precise as the haptic feedback, or as an equivalent visual image), which in our case it was not.

In what regards navigation performance, we were interested in two phenomena that acted as a proxy for participants’ learning performance. The first of these was navigation aptitude, so how well they could navigate based on their training on the tablet and in the real space, whereas the second was generalisation, so how well participants transferred what they learned from trained to untrained paths. While both groups performed relatively good on the easy trajectory, group 2 (which was trained on the easy trajectory) demonstrated difficulties when generalising from trained to untrained trajectories. Participants showed a difference in performance when tested on previously trained vs untrained trajectories, with higher accuracy and less errors on trained than untrained trajectories. Importantly, while their accuracy reached almost 90% on trained trajectories, it was drastically reduced to 60% on untrained trajectories. We ran a control t-test after checking that the Jaccard similarity indices were normally distributed within groups and did not find any significant differences between these two groups (*t*_(19.86)_ = −1.016, *p* = 0.3), indicating that both groups were able to acquire a 2D mental image of the space they learned on the tablet, which they were then able to reconstruct with LEGOs. Furthermore, given the ability of the subjects to navigate very well during the trained trajectories, this suggests that their difficulties on untrained trajectories were not due to their ability to navigate the space that they had a mental image of, so to translate this mental image into real life. A hint for a difference between the two groups might be provided by the reaction time results—namely, in group 1, participants took more time to complete trained than untrained mazes, whereas no such difference was found for group 2, which might indicate differences in how participants navigated the spaces they were previously trained on. Thus, training on a “harder” trajectory made them take more time when navigating compared to those trained on “easy” trajectories. In contrast, the group trained on “easy” trajectories (group 2) did not show any differences in reaction times between trained and untrained trajectories. Additionally, group 2 participants also spent more time off track on the untrained mazes. Indeed, group 2 did show a moderate negative correlation between IE scores and the Jaccard index on untrained trajectories, despite this correlation being non-significant after FDR correction. This might be a suggestion that a better ability to reconstruct the learned apartment layout, indexed by a high Jaccard index, was associated with better behavioural performance on these hard trajectories, which was indexed by low IE scores. While we consider the present results to be compelling, it will nonetheless be important for future research to use a wider set of trajectories to establish more fully what might be the most effective learning conditions with digital haptic technologies.

The present results further validate the potential of digital haptic technologies based on ultrasound to convey spatial information to the extent that participants can use this information to navigate real spaces. We have previously shown that digital haptic technology using ultrasounds can be successfully employed to convey spatial information and support the creation of mental images^1,2^. Specifically, sighted and visually impaired blindfolded participants were able to form mental images of 2D letters that they could then also mentally manipulate, after a similar training time with the tablet (45 minutes). Participants were trained on two letters and then tested on four letters to assess, as in the present experiment, generalisation to new stimuli. We previously found that sighted participants encountered more difficulties when tested on new letters than visually impaired participants^1,2^. Unlike for the existing study, where participants trained on harder stimuli had an easier time generalising to a new path than participants trained on an easier one, we did not observe a differential effect of group in the mental rotation task. However, it is arguably harder to parametrise how easy or hard the perception and encoding of the letters F and G versus L and P was.

The present demonstrations regarding digital haptics align with increasing interest into creating tactile displays and other digital devices to support object exploration^3^ as well as mapping and navigation^55–58^. Spatial representations related to mobility and spatial navigation can be supported by information of auditory, visual, and tactile nature^11^, and are thus arguably multisensory^59^. Despite mostly depending on visual mediation^60,61^, spatial relations can successfully be conveyed by information from other senses^8,11,16,62^, such as tactile^3,33,63–66^ or auditory information^67–70^. Haptically the shape of an object is encoded in the spatial pattern of activation evoked in mechanoreceptive fibres, much like for vision the shape of an object is encoded in the spatial pattern of activation of photoreceptors in the retina^71^. Even when vision is completely absent since birth, this does not induce any cognitive deficits in spatial navigation^72^, despite volumetric reductions in cortical regions involved in spatial tasks^73–75^, speaking in favour of a multimodal nature of spatial functions. In addition, during dynamic sensing mechanoreceptors move relative to one another as hand posture changes, thus providing a flexible and deformable sensory sheet^76^. This characteristic is particularly suited for novel digital applications capitalising on dynamic haptic perception, and for 3D shape perception in multi-touch applications^71^, such as ultrasonic airborne haptic holograms^77^.

Nevertheless, haptic perception is a complex phenomenon, which leaves large room for improvement of existing technologies. Our ability to sense characteristics such as object shape and texture likely depends on intricate sensory-motor feedback loops and on the integration of cutaneous signals from contact points with objects and proprioceptive signals about mechanoreceptor configuration^71^. The mechanoreceptors that are the most successful in coding the spatial pattern of activation for shape information are actually slow adapting type 1 (SA1) afferents, which respond to static indentations or slowly moving stimuli, whereas it is rapidly adapting (RA) fibres and Pacinian corpuscles that encode vibrations, fine textures, and movement on the skin (for a review, see ref. ^71^) thus most likely responsible for transmitting the dynamic haptic sensation that our participants were sensing here. Promisingly, however, digital haptics is a rapidly expanding field nowadays, with many research teams worldwide attempting improvements in numerous ways. For example, there are teams researching how to best transform real-world to digital textures^78^, or using haptic illusion to improve shape perception in virtual haptic displays^79^. Such detailed refinement is crucial, as spatial cognition is an intricate phenomenon. During spatial cognition not only does the perceiving individual need to sense, but also to acquire spatial knowledge based on what they sense, to be able to organise the sensory information properly, and employ it to adapt sensory and motor responses^80^.

While the present study shows the efficacy of this technology in sighted, future work should also consider applications for the blind and visually impaired. Nonetheless, it will be important to also consider the differences in haptic experience, expertise, as well as perceptual/cognitive processes during translational efforts in these subject pools. Digital haptics constitute a valuable resource for the rehabilitation of spatial functions in the visually impaired, as well as a promising tool for mobility and navigation. Vision is the predominant sense guiding our interaction with our environment, and supporting many of our everyday functions^81^. A major functional issue in the life of the visually impaired is diminished mobility, especially when diagnosed with peripheral visual field defects such as found in retinitis pigmentosa or advanced glaucoma. Mobility depends on the integrity of our spatial functions, which in turn depend on mental representations that themselves rely on the correct functioning of cortical visual mechanisms^82^. Loss of visual functions through visual impairment or blindness can affect the way that mental representations are created, which can then impair functions such as reading, manipulation of objects, or orientation in space^82–84^. Regarding navigation and orientation in space, congenital blindness is especially associated with impairments in allocentric (i.e., object-based representations; representation of an object or a space independent of one’s body or viewpoint) strategies^85^, as early visual deprivation prompts the use of body-based, egocentric spatial representations (representation of objects or spaces in relation to one’s own body)^86^, and impedes the use of an external frame of reference^87^. Moreover, the fact that in a study, controls were better than congenitally blind subjects at solving tactile multiple T mazes under restriction of environmental and proprioceptive cues^88^ indicates that digital haptics will need to be complemented by other technologies to offer a successful means of rehabilitation of the visually impaired. Indeed, current studies investigating mental map creation and wayfinding in the blind and visually impaired make use of a combination of auditory, haptic, and GPS technologies^6^. When visually impaired and blind individuals can move and orient themselves in the environment safely and autonomously, they are more likely to feel safe and independent, which is considered essential for their integration into a complex society^89^.

Besides the rehabilitation use in the visually impaired, digital haptics has already been used in other patient groups for neurorehabilitation. For example, patients who are suffering from visuospatial neglect have been shown to collide more with contralesional and head-on obstacles when navigating virtual mazes than neurologically intact individuals^90^. To this end, introducing digital haptic cues in virtual environments might alleviate these patients’ contralesional deficits. Indeed, cues delivered in another sensory modality than vision have been shown to affect orientation towards the neglected hemifield^91^ and improve detection^92^ in these patients as compared to visual cues delivered alone. In addition, digital haptics are also being used during robot-assisted training in virtual environment to provide realistic haptic rendering while supporting neurological patients to perform motor tasks^93–95^, where it has been shown to improve task performance. Specifically, sensory loss after neurological issues^96^ has been associated with poor motor recovery prognosis^97^. Despite current inconclusive results regarding their effectiveness^98^, researchers now design numerous haptic training methods to support motor learning with robots^99^. Nevertheless, besides sensory learning, haptics has also been associated with increased motivation during motor training^95^, speaking in favour of their continued use in rehabilitation approaches.

In conclusion, digital haptics can successfully convey spatial information from which individuals can constitute mental topographic maps that they can use to efficiently navigate real-world spaces. This has important implications for sensory substitution and numerous applications in the fields of neurorehabilitation—such as rehabilitation of lost functions after visual loss, or when suffering from visuospatial neglect, but also motor rehabilitation and virtual reality applications. Continued refinement and research into this technology could improve its efficacy and enlarge its horizon of application towards new fields—such as education and leisure. This is an endeavour that our team, as well as others around the globe are currently pursuing with high excitement.

## Methods

### Participants

All participants provided written informed consent to procedures approved by the cantonal ethics committee (protocol 2018-00240). We tested 25 adults (15 women and 10 men; age range 18-39 years, mean ± stdev: 27.08 ± 4.04 years), who volunteered for our experiment. Participants reported normal or corrected-to-normal vision. No participant had a history of or current neurological or psychiatric illness. Handedness was assessed via the Short Form of the Edinburgh Handedness Inventory^100^. One of our participants was left-handed, while the remainder were right-handed.

### Apparatus

Haptic stimulation was delivered via a tablet with a TFT capacitive 7-inch touchscreen with a resolution of 1024 × 600 pixels. The screen of the tablet was controlled by a Raspberry Pi 3-based system, and the operating system was Raspbian (Linux). The processor of the tablet was a Broadcom ARMv7, quadcore 1.2 GHz, and it had 1 Go RAM and Rev C WaveShare. The tablet came with a haptic creation tool, which is software that allows for user control of haptic textures. Several other APIs based on C++ or Java were installed, such as library tools that allow the implementation of haptics on other applications. Figures in jpeg format were re-coded in haptic format using a kit written in C++. For more technical details describing the rendering of the haptic feedback, see refs. ^101–103^.

**Fig. 5:**
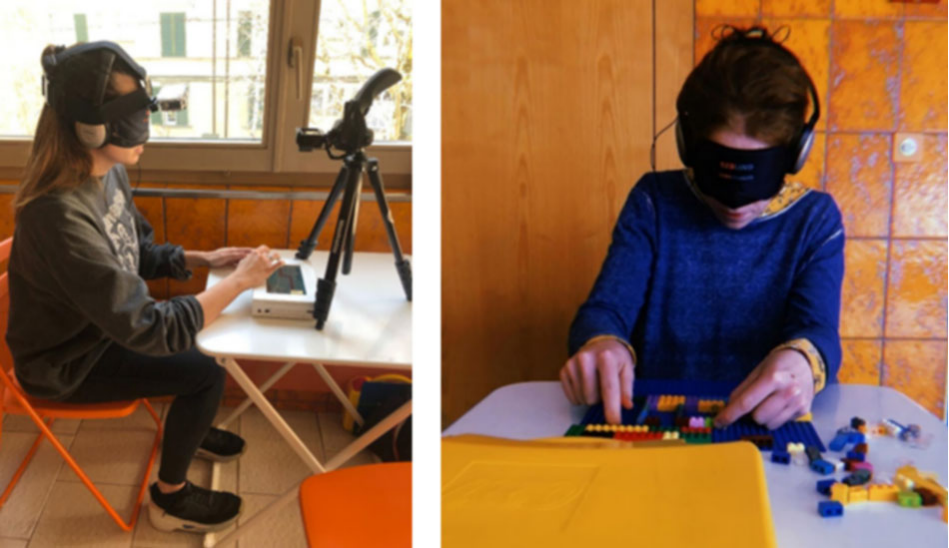
Experimental setup (left) and LEGO^®^ reconstruction (right). Participants were blindfolded and wearing noise-cancelling headphones while interacting with the tablet. They were wearing a GoPro camera mounted on their head which filmed their path during the navigation part of the experiment. Another GoPro filmed their tablet exploration (data not analysed here). On the right, the photo shows a participant reconstructing the learned background layout while blindfolded and wearing headphones. Written consent was obtained for the publication of these photographs.

The performance of the participant inside the apartment was filmed with a GoPro 4 Silver strapped to a headband that the participant was wearing over the noise-cancelling headphones. Another GoPro 5 Black was used to film the exploration hand of the participant, to analyse exploration strategies and how these change as a function of training. An illustration of the setup can be seen in Fig. 5. Individual consent was obtained for the publication of these photographs. These data (i.e., GoPro videos) are not presented in the current manuscript.

### Stimuli

Stimuli consisted of three images in jpeg format that had been created in Paint based on the floor plan of the living lab apartment (Fig. 1). Image size was 1023 × 574 pixels. Images depicted the floorplan: one of them did not include any trajectories, and the other two depicted two different trajectories throughout the apartment. These haptic maps consisted of four different textures, which were designed and chosen together with occupational therapists who work with visually impaired people on rehabilitation of their spatial and mobility skills (i.e., locomotion training with real texture maps). The four different textures represented either (1) walls, with a break in the line of the wall being associated with a door, (2) furniture (i.e., obstacles), (3) areas outside of the apartment (i.e., which had to be represented on the floor plan in order to respect proportions), or (4) the trajectory itself. Regarding the image-to-haptic conversion, the different features of the floorplan appeared on a white background. White pixels did not result in a texture on the finger. All non-white pixels of the floorplan were then coded with three different haptic textures, which were created using the hap2u pre-installed Texture Editor software. The ultrasonic vibration was adjusted to have different shapes: a square shape (walls, areas outside, and obstacles), as this offers the most intense and quick reduction of the friction of the screen under the finger, thus conferring a rather sharp and pointy sensation; this was contrasted to a sinusoidal-shaped wave that was used for the trajectory, which confers a rather smooth perception. We used these two ultrasonic wave shapes, as they are provided in the Haptic Texture Library Creation Tool that is native to the device. The period of the window of one square ultrasonic signal was chosen to be 4660 µm (which is considered a “coarse” texture, see Hollins & Risner, 2000), and for the sinusoidal signal, it was 2550 µm. The amplitude was always set at 100%, meaning ~2 µm (as the friction reduction hits a plateau at this value, see, e.g., Sednaoui et al., 2017).

### Procedure and task

Participants were trained in a corridor exterior to the living laboratory (apartment). The testing phase occurred inside the apartment (see Fig. 2 for a schematic illustration of the procedure). Subjects were blindfolded and wore noise-canceling headphones (Bose model QuietComfort 2) during the entire duration of the experiment (i.e., training and testing), in order to block any residual light and any potential sounds of the ultrasonic vibrations produced by the digital haptics tablet. None of the participants had any prior visual or haptic exposure to the stimuli used in the paradigm, minimising any cross-modal facilitation^104^, as would be the case if participants were able to use the sounds produced by the haptic tablet to localise their fingers on the tablet.

The participant’s task was comprised of multiple parts. Participants had to feel a haptic rendering of a map of the living lab apartment on the tablet screen. Subjects were instructed to use a finger from their dominant hand for tablet exploration, and to hold the non-dominant hand on the side of the tablet to serve as a spatial checkpoint, by which participants could map the extent of the tablet, or its size, with one hand, while exploring its screen with the other. The task was to feel the map, which was a haptic rendering of a jpeg image of the living laboratory apartment’s floorplan. After becoming familiarised with the different textures and the main layout map (see next paragraph for a description of this procedure), participants had to reconstruct the map in LEGO® bricks, while keeping their blindfolds on. Then, participants were trained on one of two possible trajectories in the apartment. Half of the participants were assigned randomly and in a counterbalanced manner to be trained on a trajectory leading to Room1 (i.e., bedroom), and the other half on a trajectory leading to Room 2 (i.e., kitchen). The layout of the space can be seen in Fig. 1, where the rooms and the error zones are annotated. We chose these two rooms as they seemed to require the same strategical difficulty. From the entrance of the living lab apartment, participants had to take two turns to reach either of these target rooms, while in order to reach any other of the rooms, the strategy employed would have had to be different. We decided to focus the training on a particular trajectory to investigate skill transfer to a new, untrained trajectory. The decision to use only two rooms out of the four emerged after practical observations: the duration of the procedure was already roughly two hours.

During the training phase, participants were first trained to explore the tablet screen via lateral sweeps of their finger^17^, see e.g., ^105^ for a discussion of which tactile exploration strategies are particularly appropriate to disclose specific object characteristics, and^106^ for a discussion of how dynamic vs. static exploration affects coarse (>100 µm) as compared to fine texture discrimination). Subjects were allowed to change the finger they used for exploration, due to a common remark about adaptation of their tactile sensation during the pilot experiments or during the training blocks. However, they were not allowed to change the hand used for exploration. Subjects were then taught how to discriminate between different textures and their associations, and finally, how to recognise rooms and trajectories in the map going from the entrance to one of the four rooms. The experimenter gave subjects verbal instructions and verbal feedback throughout the training session regarding these aforementioned aspects, but not about successful exploration.

At the end of the training session, participants had to verbally describe the background map to the experimenter, ensuring that the main layout and the number of rooms, as well as the shape of the corridor (i.e., spaces between the rooms and turning points in the corridor) were correctly understood, which was assessed through verbal reports. When this criterion was reached, i.e., when participants reported the correct number of rooms and their placement, as well as correctly identified the shape of the corridor running between the rooms, participants could pass to the reconstruction stage of the procedure (see Fig. 2). This stage involved using LEGO® pieces on a LEGO® board while still being blindfolded. The LEGO® board was adjusted and constructed to respect the layout of the apartment (see Fig. 3a). Participants had the following pieces at their disposal: 41 pieces of 1*2 units, 2 pieces of 1*3 units, 4 pieces of 1*4 units, and 2 pieces of 1*6 units. Their task was to reconstruct the layout of the apartment as accurately as possible. Not all pieces were required to complete the reconstruction. Participants were not explicitly informed of this and were instructed to use the pieces they considered necessary for an accurate reconstruction.

After reconstructing the space in the LEGO® task, participants were introduced to the trajectory texture on the digital haptics tablet, and haptic training on a certain trajectory in the previously learned 2D space continued. When they reported having understood the trajectory, participants were taken inside the apartment, where they were first familiarised with sightless exploration and navigation techniques of this space. While attempting to carry out the trained trajectory (still blindfolded), participants learned about error zones, and what would be counted as an error during the testing phase. During this phase, participants were not instructed on where to go and did not receive any feedback on whether their movements were accurate, or whether they were taking the correct direction, or whether they successfully completed the trained trajectory.

After this short familiarisation with sightless navigation, the testing phase began, where participants were instructed to independently explore the tablet and then physically carry out the presented trajectory. The testing phase was comprised of 10 trials, making 5 trials in total per participant per trajectory, in which Room1 and Room2 were randomly presented. Participants were instructed to explore the tablet for as long as it took for them to be sure of the trajectory on each trial, without a time limit being enforced for this stage. They were, however, timed. When they verbally reported being sure of the trajectory, participants were instructed to be as quick and as accurate as possible in navigating this trajectory. Errors were scored during path deviations to pre-defined error zones. Points were given according to how far participants deviated into error zones. The time that participants spent “off track” was also measured. This measure represents the time in seconds that the participant spent off the trajectory they were supposed to take, i.e., the time spent while doing errors or while being in the error zones. If participants did not reach the target room, the specific trial was scored as “Missed”. The numbers in Fig. 1 represent the scoring for error zones, e.g., if someone goes through the door at the end of the first hallway, their error score is 0.5 points. If they just touch the door or walk into it (in order to find out that the corridor ends), they earn 0.25 error points. This is because they used this landmark for orientation when they are not allowed to. Errors were scored independently of accuracy. It was possible to have accuracy = 1 and an error scores unequal to zero. It was impossible to have accuracy = 0 and error points. In “Missed” trials, errors did not get scored. As soon as the participant entered the error zones, they got scored the associated error points, as noted in the pictures. When they finished, participants were then taken to the entrance to the apartment by the experimenter. A GoPro filmed participants’ performance inside the apartment. The experimenter then took them back to the table where the tablet was located to proceed with the next trial. During the experiment, participants were allowed to take regular breaks between trials to maintain high concentration and prevent fatigue. Stimulus delivery and behavioural response collection were controlled by the experimenter, using an iPhone 8 Timer Application for timing purposes.

### Inter-rater reliability

To validate the Jaccard index, we asked three independent raters to subjectively rate the photos of the LEGO® reconstructions (i.e., how well the reconstructions compared to an ideal reconstruction), using a scale from 1 to 5. These ratings were checked for agreement using inter-rater reliability (IRR^48^). Given that behavioural performance was also assessed by multiple independent raters scoring videos, we applied IRR to these ratings as well. Cohen’s kappa values were computed for assessing inter-rater reliability of Accuracy scores, as these data are nominal, whereas intraclass Correlation (ICC), which is better suited to ordinal and ratio scales, was used to assess consensus regarding scoring of Errors and of the Time off-track.

### Image analysis

Images were first manually processed through Photoshop CS5, to apply a 3D warping (i.e., 3D rotation and resize), to correct for the different angles the pictures from which the photos were taken by the experimenter. We would note that this step will not be necessary for the future, as a standardised way to take these pictures has subsequently been identified. The pre-processed images were then imported in Matlab R2019b. Images were processed first by applying a chromatic adaptation step^96^, to rebalance the three red, green, and blue channels, by choosing as a reference a background blue pixel. Afterwards the histogram equalisation was applied to enhance the contrast of the images. Due to the presence of blue salient LEGO® over a blue background, the difference between the first and last equalised channels was computed and binarized. The binarized images were then converted to double, and the common parts among all figures were flattened. Morphological operations of opening and closing were then applied to first remove noisy pattern of circles (1 pixel radius) around the image, then to enhance squared structures (10 pixels edge). Every image *I* was compared with the ground truth representation *B* of the maze by means of the Jaccard index, a similarity index that evaluates the intersection above the union of two sets of sample points:

1 $$J\left(B,I\right)=\frac{\left|B\cap I\right|}{\left|B\cup I\right|}$$

The mean subjective ratings had the role of validating that the Jaccard was a representative measure of participants’ reconstructions. To this aim, we first checked whether their distributions followed a normal distribution using Shapiro-Wilk tests. After confirmation of distributional properties, we ran a correlation analysis using Pearson’s *r* to check whether the ratings were significantly correlated with the Jaccard Index.

### Behavioural analysis

Data were analysed in R (R Core Development Team, 2017) and Matlab (Mathworks, v. R2017a). Mean behavioural scores were recalculated according to the mean of both ratings. These mean scores were used in the final analysis. From these scores, means were calculated for Accuracy (Acc), RT, Tablet Exploration Time (ET), Errors (E), and Time off track (Toff). Only accurate trials were used for the analysis of RT, ET, E, and Toff. Pilot results indicated a significant difference between performance of participants on the two trajectories, and pilot participants reported feeling that one trajectory was more difficult than the other. Specifically, pilot results indicated that participants took longer to arrive at Room1 (30.7 s) than Room2 (16.4 s), despite the trajectory lengths being very similar (Room1: 11.3 m vs. Rooms 2: 10.96 m), and with participant accuracies for both trajectories being equal (i.e., 80% correct). We therefore included Group (Group 1: trained on the harder trajectory, vs. group 2: trained on the easier trajectory) as a factor in our analyses. See ref. ^107^ for a parametrization of how a maze path can influence navigation in mice. We further calculated Inverse Efficiency scores (IE^108^). IEs constitute a standard approach to combine RT and accuracy measures of performance and can be considered “corrected reaction times” that discount possible criterion shifts or speed/accuracy trade-offs (see Fig. 4). We compared means with a 2 × 2 permutation repeated-measures mixed-design ANOVA with the within-subjects factor TRAINING (trained vs untrained) and between-subject factor GROUP, after having found significant deviations from the normal distribution of the residuals.

### Reporting summary

Further information on research design is available in the Nature Research Reporting Summary linked to this article.

### Supplementary information

Reporting Summary

## Data Availability

The datasets supporting the findings reported here may be shared upon request for research purposes, provided the request is in line with current privacy regulations.
