# Supplementary material for: Learning and navigating digitally rendered haptic spatial layouts
Source: NPJ Sci Learn. 2023 Dec 16;8:61. doi: 10.1038/s41539-023-00208-4 (PMC10724186; doi:10.1038/s41539-023-00208-4)
Supplement: Supplementary file 1 — Reporting Summary [file 41539_2023_208_MOESM1_ESM.pdf]

nature portfolio

Corresponding author(s): Micah Murray

Last updated by author(s): 08/03/2023

Reporting Summary

Nature Portfolio wishes to improve the reproducibility of the work that we publish. This form provides structure for consistency and transparency in reporting. For further information on Nature Portfolio policies, see our Editorial Policies and the Editorial Policy Checklist.

Statistics

For all statistical analyses, confirm that the following items are present in the figure legend, table legend, main text, or Methods section.

n/a	Confirmed	
☐	☒	The exact sample size (*n*) for each experimental group/condition, given as a discrete number and unit of measurement
☐	☒	A statement on whether measurements were taken from distinct samples or whether the same sample was measured repeatedly
☐	☒	The statistical test(s) used AND whether they are one- or two-sided<br>*Only common tests should be described solely by name; describe more complex techniques in the Methods section.*
☒	☐	A description of all covariates tested
☐	☒	A description of any assumptions or corrections, such as tests of normality and adjustment for multiple comparisons
☐	☒	A full description of the statistical parameters including central tendency (e.g. means) or other basic estimates (e.g. regression coefficient) AND variation (e.g. standard deviation) or associated estimates of uncertainty (e.g. confidence intervals)
☐	☒	For null hypothesis testing, the test statistic (e.g. *F*, *t*, *r*) with confidence intervals, effect sizes, degrees of freedom and *P* value noted<br>*Give P values as exact values whenever suitable.*
☒	☐	For Bayesian analysis, information on the choice of priors and Markov chain Monte Carlo settings
☒	☐	For hierarchical and complex designs, identification of the appropriate level for tests and full reporting of outcomes
☐	☒	Estimates of effect sizes (e.g. Cohen's *d*, Pearson's *r*), indicating how they were calculated

Our web collection on statistics for biologists contains articles on many of the points above.

Software and code

Policy information about availability of computer code

Data collection	All methods are detailed in the manuscript and computer / software code used for data collection are described or information is provided to readers on where such can be obtained.
Data analysis	Analyses have been detailed in the Methods section and include the use of open source softwares such as R.

For manuscripts utilizing custom algorithms or software that are central to the research but not yet described in published literature, software must be made available to editors and reviewers. We strongly encourage code deposition in a community repository (e.g. GitHub). See the Nature Portfolio guidelines for submitting code & software for further information.

Data

Policy information about availability of data

All manuscripts must include a data availability statement. This statement should provide the following information, where applicable:

- Accession codes, unique identifiers, or web links for publicly available datasets
- A description of any restrictions on data availability
- For clinical datasets or third party data, please ensure that the statement adheres to our policy

The datasets generated during and/or analyzed during the current study are available from the corresponding authors on reasonable request.

Research involving human participants, their data, or biological material

Policy information about studies with human participants or human data. See also policy information about sex, gender (identity/presentation), and sexual orientation and race, ethnicity and racism.

Reporting on sex and gender	In the participants description, we reported sex based on self-report, but it was not a prerequisite for inclusion to the study. For the same reason, we did not perform any sex-based or gender-based analysis, as the outcome of the present study does not relate to either sex or gender.
Reporting on race, ethnicity, or other socially relevant groupings	Any socially constructed or socially relevant categorization variable was not used nor relevant to the outcome of the present study.
Population characteristics	The population of the present study is composed by 25 healthy individuals between 18 and 39 years, with no previous medical history (of any type), as determined by the questionnaire approved by the local IRB.
Recruitment	Participants were recruited among university students via online announcements. Subjects were chosen on a first-come first-served basis. We verified that none of the selected subjects shown hierarchical link to the researcher or any other author of the paper. All participants signed an informed consent before undergoing the study, where they were asked to state the absence of previous and current medical conditions.
Ethics oversight	The study protocol received approval by the cantonal ethics (CER-VD) committee (protocol number 2018–00240)

Note that full information on the approval of the study protocol must also be provided in the manuscript.

Field-specific reporting

Please select the one below that is the best fit for your research. If you are not sure, read the appropriate sections before making your selection.

☐ Life sciences ☒ Behavioural & social sciences ☐ Ecological, evolutionary & environmental sciences

For a reference copy of the document with all sections, see nature.com/documents/nr-reporting-summary-flat.pdf

Behavioural & social sciences study design

All studies must disclose on these points even when the disclosure is negative.

Study description	This is a study in health adult humans of learning spatial layouts based solely on touch, reproducing them with LEGOs and then actually navigating in the learned space. We used quantitative metrics on the LEGO reconstruction and movement in the space.
Research sample	We studied healthy adult volunteers recruited via printed flyers and social media, pedominantly from the Lausanne University community. Participants were aged 18-39 years (15 women and 10 men).
Sampling strategy	The sampling procedure was convenience. The sample size was based partially on prior studies as well as our own power analysis (details of which appear in our replies to reviewers).
Data collection	Participants were blind to the study hypotheses during data collection. Researchers were not themselves blinded to the objectives of the study. Data collection was performed with a digital haptics tablet provided by hap2u (www.hap2u.net), video recording devices (GoPro camers), and LEGO blocks.
Timing	Data were collected between January and July of 2019
Data exclusions	no participants were excluded.
Non-participation	not applicable.
Randomization	The two groups were randomized based on a random number seed

Reporting for specific materials, systems and methods

We require information from authors about some types of materials, experimental systems and methods used in many studies. Here, indicate whether each material, system or method listed is relevant to your study. If you are not sure if a list item applies to your research, read the appropriate section before selecting a response.

Materials & experimental systems

n/a	Involved in the study
☒	☐ Antibodies
☒	☐ Eukaryotic cell lines
☒	☐ Palaeontology and archaeology
☒	☐ Animals and other organisms
☒	☐ Clinical data
☒	☐ Dual use research of concern
☒	☐ Plants

Methods

n/a	Involved in the study
☒	☐ ChIP-seq
☒	☐ Flow cytometry
☒	☐ MRI-based neuroimaging
